# Amyloid-β (Aβ) immunotherapy induced microhemorrhages are linked to vascular inflammation and cerebrovascular damage in a mouse model of Alzheimer’s disease

**DOI:** 10.1186/s13024-024-00758-0

**Published:** 2024-10-21

**Authors:** Xavier Taylor, Harun N. Noristani, Griffin J. Fitzgerald, Herold Oluoch, Nick Babb, Tyler McGathey, Lindsay Carter, Justin T. Hole, Pascale N. Lacor, Ronald B. DeMattos, Yaming Wang

**Affiliations:** 1 Lilly Research Laboratories, Neuroscience Discovery, Eli Lilly and Company, Indianapolis, 46225 IN USA; 2 Lilly Research Laboratories, Imaging Research and Development, Eli Lilly and Company, Philadelphia, PA 19107 USA

## Abstract

**Background:**

Anti-amyloid-β (Aβ) immunotherapy trials have revealed amyloid-related imaging abnormalities (ARIA) as the most prevalent and serious adverse events linked to pathological changes in cerebral vasculature. Recent studies underscore the critical involvement of perivascular macrophages and the infiltration of peripheral immune cells in regulating cerebrovascular damage. Specifically, Aβ antibodies engaged at cerebral amyloid angiopathy (CAA) deposits trigger perivascular macrophage activation and the upregulation of genes associated with vascular permeability. Nevertheless, further research is needed to understand the immediate downstream consequences of macrophage activation, potentially exacerbating CAA-related vascular permeability and microhemorrhages linked to Aβ immunotherapy.

**Methods:**

This study investigates immune responses induced by amyloid-targeting antibodies and CAA-induced microhemorrhages using RNA in situ hybridization, histology and digital spatial profiling in an Alzheimer's disease (AD) mouse model of microhemorrhage.

**Results:**

In the present study, we have demonstrated that bapineuzumab murine surrogate (3D6) induces profound vascular damage, leading to smooth muscle cell loss and blood–brain barrier (BBB) breakdown. In addition, digital spatial profiling (DSP) reveals that distinct immune responses contribute to vascular damage with peripheral immune responses and perivascular macrophage activation linked to smooth muscle cell loss and vascular fibrosis, respectively. Finally, RNA in situ hybridization identifies two distinct subsets of *Trem2*^+^ macrophages representing tissue-resident and monocyte-derived macrophages around vascular amyloid deposits. Overall, these findings highlight multifaceted roles of immune activation and vascular damage in driving the development of microhemorrhage.

**Conclusions:**

In summary, our study has established a significant link between CAA-Aβ antibody immune complex formation, immune activation and vascular damage leading to smooth muscle cell loss. However, the full implications of this cascade on the development of microhemorrhages requires further exploration. Additional investigations are warranted to unravel the precise molecular mechanisms leading to microhemorrhage, the interplay of diverse immune populations and the functional roles played by various *Trem2*^+^ macrophage populations in response to Aβ immunotherapy.

**Supplementary Information:**

The online version contains supplementary material available at 10.1186/s13024-024-00758-0.

## Introduction

Deposition of amyloid-beta (Aβ) aggregates in the brain parenchyma and neurovascular space is the defining feature of Alzheimer’s disease (AD) pathology [1]. Passive immunotherapy, which aims to remove toxic amyloid deposits, has emerged as one of the most promising strategies in combating AD. Several large late-stage clinical trials have substantiated the success of this strategy, showcasing significant improvements in soluble biomarkers, reduction of amyloid pathology, and cognitive decline [2–4] validating the long-debated amyloid cascade hypothesis [5]. Despite these favorable outcomes, amyloid-related imaging abnormalities (ARIA), a spectrum of radiological imaging findings consistent with brain edema (ARIA-E) and microhemorrhages (ARIA-H), persist as the most common adverse events reported in these trials [6, 7], restricting implementation of these promising therapies to the broader patient population. Importantly, the precise mechanisms by which Aβ immunotherapy induces vascular permeability, blood–brain barrier (BBB) breakdown, and subsequent microhemorrhages remain elusive. Neuropathological evidence from Aβ immunotherapy patients supports the occurrence of perivascular inflammation, characterized by reactive perivascular macrophages (PVM) engaged in Aβ phagocytosis and clearance within cerebral blood vessels, emphasizing the integral role of macrophages in the host response to Aβ immunotherapy [7, 8]. Notably, we have previously demonstrated immune complexes formation between Aβ antibodies and vascular amyloid deposits triggers activation of PVMs, underscoring their critical role in initiating vascular inflammation damage during Aβ immunotherapy [9]. Interestingly, recent data from INVOKE-2 phase II study revealed a marked increase in ARIA events after TREM2-agonist antibody treatment [10], suggesting that activation of macrophage lineage cells without directly engaging amyloid deposits is sufficient to trigger ARIA in AD patients. To comprehensively profile the dynamics of vascular inflammation and damage leading to ARIA, we employed a well-established mouse model of Aβ immunotherapy-induced microhemorrhages, to elucidate the immune responses and vascular damage linked with vascular permeability, plasma protein extravasation, and microhemorrhages [11].

Herein, our results demonstrate that Aβ immunotherapy in PDAPP mice triggers profound changes in the vascular inflammatory microenvironment and cerebrovascular damage characterized by the loss of smooth muscle cells and vascular fibrosis. Additionally, we observe a prominent upregulation of vascular endothelial growth factor (VEGF), astrocytic aquaporin-4 (AQP4) and glial fibrillary acidic protein (GFAP) at the glia limitans, which potentially function as a compensatory mechanism. Spatial proteomics further reveals a multifaceted immune profile, particularly in the leptomeninges, highlighting PVM responses associated with vascular fibrosis and peripheral immune responses linked to smooth muscle cell loss. Importantly, our study demonstrates that two distinct subsets of *Trem2*^+^ tissue-resident and monocyte-derived macrophages are highly associated with vascular amyloid deposits following Aβ immunotherapy. This finding establishes a mechanistic connection between Aβ immunotherapy, vascular inflammation, compromised BBB integrity, and the recruitment of peripheral immune cells to vascular amyloid deposits.

## Results

### Aβ immunotherapy triggers vascular damage, smooth muscle cell loss and disrupted BBB permeability

**Fig. 1:**
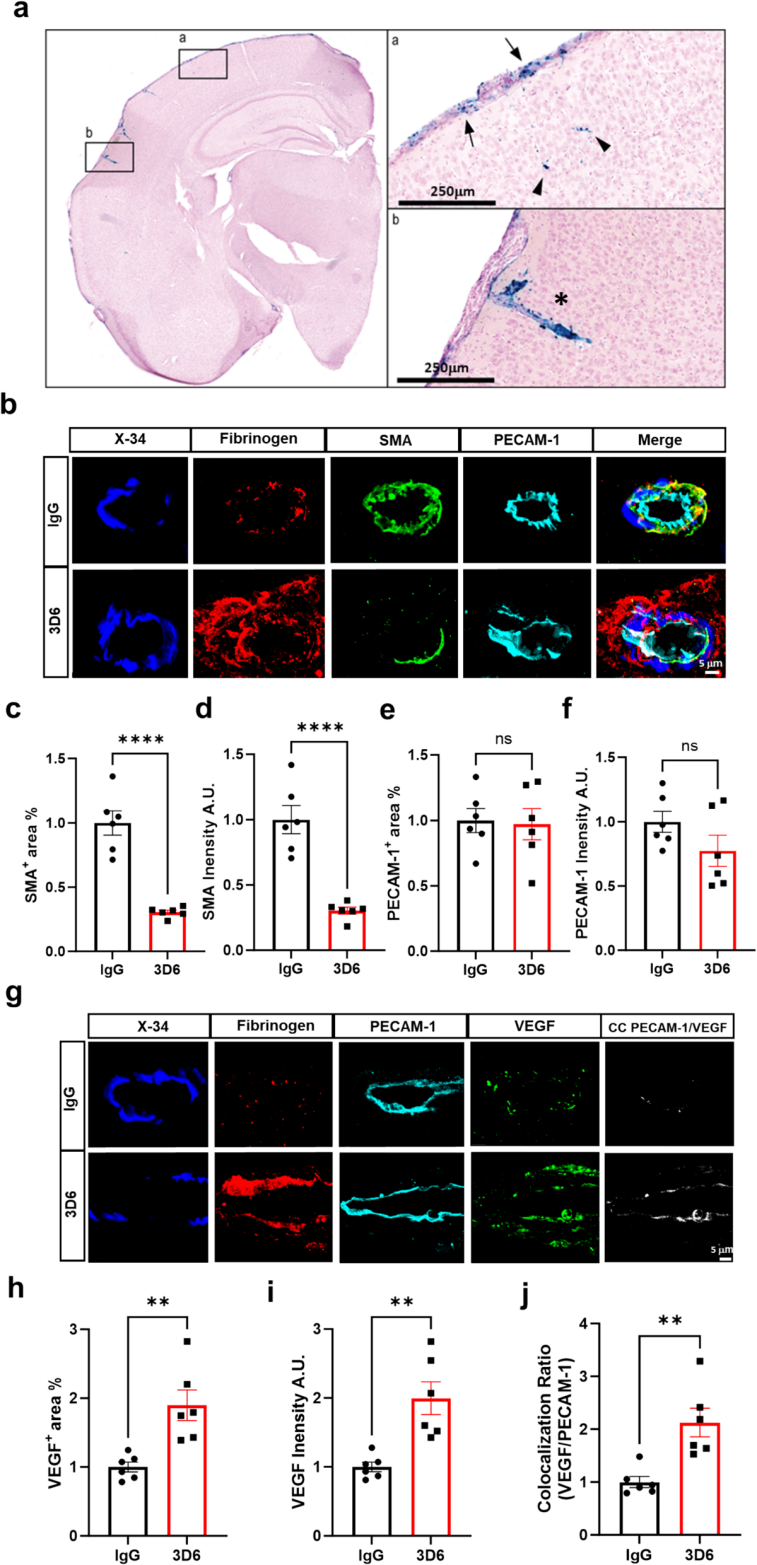
3D6 treatment induces profound smooth muscle cell loss in PDAPP mice. **a** Prussian Blue (hemosiderin, blue) labeled microhemorrhages across leptomeninges (arrow), parenchyma (triangle) and penetrating vessels (asterisk) of PDAPP mice treated with 3D6. **b** Four-color immunofluorescence of amyloid (X-34, blue), fibrinogen (red), smooth muscle cells (SMA, green) and endothelial cells (PECAM-1, cyan) in PDAPP mice treated with 3D6 or IgG control. X-34, Fibrinogen, SMA and PECAM-1 immunoreactivity overlay (Merge). **c** Quantification of SMA^+^ area (%) of IgG or 3D6 treated PDAPP mice. **d** Quantification of intensity of SMA in IgG or 3D6 treated PDAPP mice. **e** Quantification of PECAM-1^+^ area (%) of IgG or 3D6 treated PDAPP mice. **f** Quantification of intensity of PECAM-1 in IgG or 3D6 treated PDAPP mice. The number of vascular amyloid deposits analyzed was 8–10 per animal. Results are shown as mean ± SEM of subgroup analysis *n* = 6 (mice). **g** Four-color immunofluorescence of amyloid (X-34, blue), fibrinogen (red), endothelial cells (PECAM-1, cyan) and vascular endothelial growth factor (VEGF, green) in PDAPP mice treated with 3D6 or IgG control. X-34, Fibrinogen, PECAM-1 and VEGF immunoreactivity overlay (Merge). **h** Quantification of VEGF^+^ area (%) of IgG or 3D6 treated PDAPP mice. **i** Quantification of intensity of VEGF in IgG or 3D6 treated PDAPP mice. **j** Quantification of colocalization ratio of VEGF and PECAM-1. The number of vascular amyloid deposits analyzed was 8–10 per animal. Results are shown as mean ± SEM of subgroup analysis *n* = 6 (mice). All are representative images of 26-month-old PDAPP mice. Asterisks indicate significant differences, where **p < 0.01 and ****p < .0001 by unpaired Student’s t test. Scale bar 5 or 250 μm, respectively

In PDAPP mice, the leptomeninges are the primary site of vascular amyloid accumulation and show significant microhemorrhages in response to anti-Aβ (3D6) immunotherapy (Fig. 1, a) [11]. The observed pattern of vascular bleeding closely resembles nontraumatic convexity subarachnoid hemorrhage (cSAH), a noteworthy subtype of non-aneurysmal subarachnoid bleeding increasingly acknowledged as a significant imaging feature in the context of cerebral amyloid angiopathy (CAA) and ARIA [12]. Therefore, we aimed to determine the impact of anti-Aβ immunotherapy on vascular damage in 23-month-old PDAPP animals, specifically exploring areas surrounding vascular amyloid accumulation, plasma protein extravasation, and microhemorrhages. To verify the efficacy of Aβ immunotherapy in inducing microhemorrhages, PDAPP mice were treated for three months with either 3D6 or an IgG control antibody. Our findings demonstrate a significant increase in the Prussian blue^+^ area, indicating a significant increase of microhemorrhages and an expansion of leptomeningeal vessel diameter in mice treated with 3D6 compared to the control IgG group (Supplemental Fig. 1, a-c) aligning with our previous findings [9]. A significant inverse correlation was observed between microhemorrhage severity and vascular amyloid burden (Supplemental Fig. 1, d-e). To further assess vascular changes, a subset of 6 animals from the total microhemorrhage groups were chosen at random for further assessment and subgroup analysis. Consistently, we observed a reduction in vascular amyloid burden in a subset of 3D6-treated PDAPP mice, with amyloid deposits appearing more fragmented, although the reduction in parenchymal plaques was less pronounced (Supplemental Fig. 1, h-j).

To evaluate plasma protein extravasation and acute BBB leakage, we stained for fibrinogen, a coagulation protein that is absent in the vascular compartment unless there is a breach of the cerebral vasculature permeability barrier. Our data reveal the presence of plasma protein extravasation in 3D6-treated PDAPP mice, indicated by significant increases in fibrinogen^+^ area in the cortical leptomeninges compared to control IgG group (Supplemental Fig. 1, f-g).

**Fig. 2:**
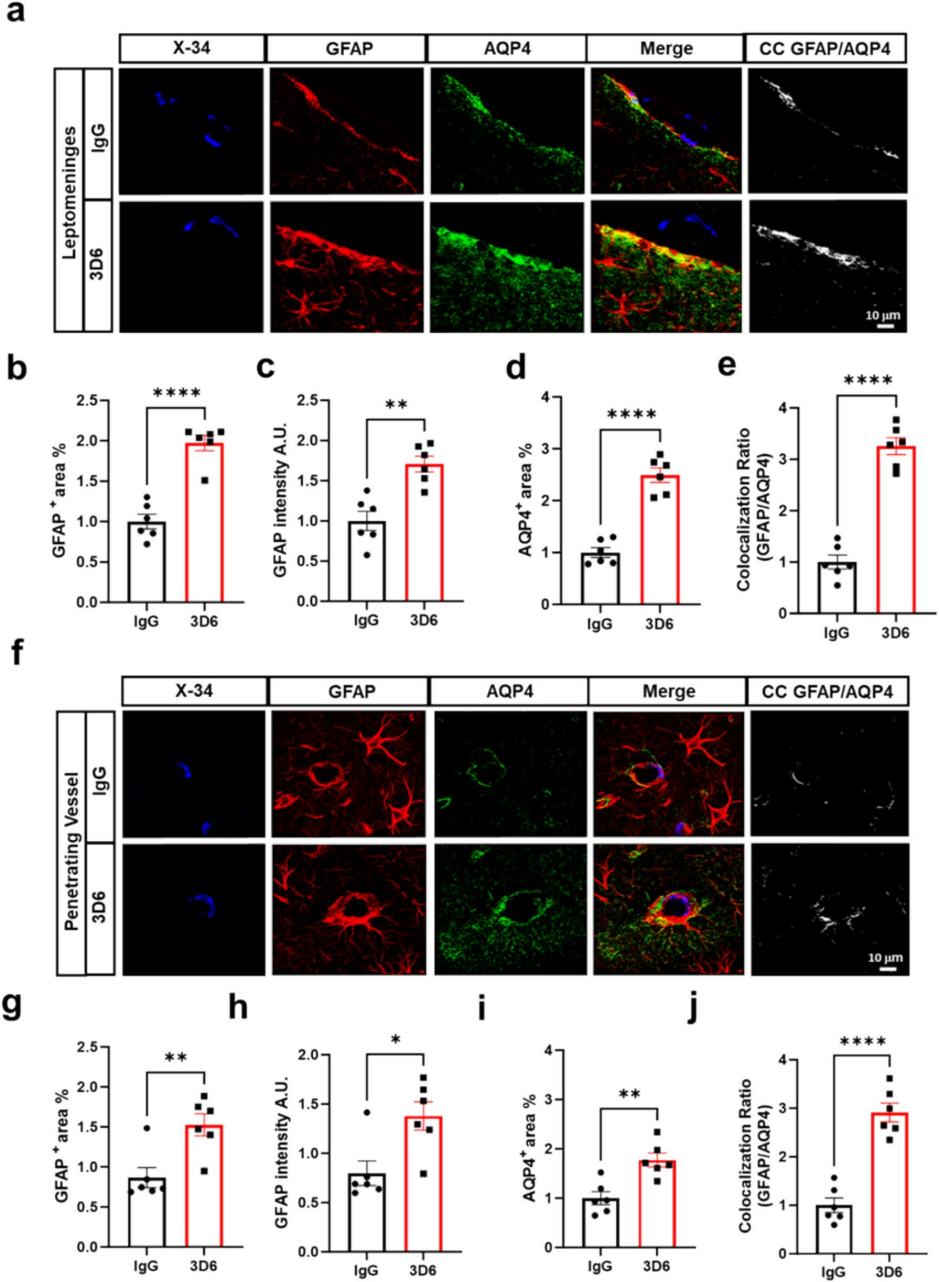
Amyloid immunotherapy is highly associated with increased astrocytic aquaporin-4 in 3D6 treated PDAPP Mice. **a** Triple immunofluorescence of amyloid (X-34, blue), astrocytes (GFAP, red) and aquaporin-4 (AQP4, green) in the leptomeninges of PDAPP mice treated with 3D6 or IgG control. X-34, GFAP and AQP4 immunoreactivity overlay (Merge). Colocalization analysis (white). **b** Quantification of GFAP^+^ area (%) of IgG or 3D6 treated PDAPP mice. **c** Quantification of GFAP intensity of IgG or 3D6 treated PDAPP mice. **d** Quantification of AQP4^+^ area (%) of IgG or 3D6 treated PDAPP mice. **e** Quantification of colocalization ratio of GFAP and AQP4. **f** Triple immunofluorescence of amyloid (X-34, blue), astrocytes (GFAP, red) and aquaporin-4 (AQP4, green) in penetrating vessels of PDAPP mice treated with 3D6 or IgG control. X-34, GFAP and AQP4 immunoreactivity overlay (Merge). Colocalization analysis (white). **g** Quantification of GFAP^+^ area (%) of IgG or 3D6 treated mice. **h** Quantification of GFAP intensity of IgG or 3D6 treated PDAPP mice.** i** Quantification of AQP4^+^ area (%) of IgG or 3D6 treated PDAPP mice. **j** Quantification of colocalization ratio of GFAP and AQP4. The number of vascular amyloid deposits analyzed was 8–10 per animal. Results are shown as mean ± SEM of subgroup analysis *n* = 6 (mice). Asterisks indicate significant differences, where *p < 0.05, **p < 0.01 or ****p < 0.0001 by unpaired Student’s t test. Scale bar 10 μm

To investigate aspects of cerebrovascular damage following Aβ immunotherapy, we evaluated the vascular integrity of BBB-associated cells utilizing X-34, fibrinogen, smooth muscle actin (SMA), and platelet/endothelial cell adhesion molecule-1 (PECAM-1). Our results show Aβ immunotherapy leads to loss of vascular smooth muscle cells with a significant decrease in SMA^+^ area and intensity compared to control IgG (Fig. 1, b-d). Additionally, no significant differences are observed in PECAM-1^+^ area and intensity (Fig. 1, e–f), or in Claudin-5^+^ area and intensity (Supplementary Fig. 2, a-c), relative to the control IgG. These results are in line with the recorded development of CAA cases, which show preservation of endothelial cells even in cases of severe CAA and degeneration of smooth muscle cells as the disease progresses [13, 14]. To further understand BBB permeability, we assessed VEGF, a secreted mitogen crucial for regulating angiogenesis, vascular permeability, and the growth and survival of endothelial cells [15]. Interestingly, our results show a significant increase in VEGF^+^ area, intensity, and colocalization ratio with PECAM-1^+^ endothelial cells in 3D6 treated PDAPP mice relative to control IgG (Fig. 1, g-j). Apart from its role in angiogenesis, VEGF is also a potent permeability factor upregulating AQP4 mRNA and protein expression in the perivascular space and glia limitans [16]. Considering this, we assessed astrocytic AQP4 in the leptomeninges and penetrating vessels in PDAPP mice following Aβ immunotherapy. Our results show in the leptomeninges increased astrocytic GFAP^+^ area and intensity are highly associated with vascular amyloid deposits in 3D6 treated PDAPP mice (Fig. 2, a-c). This is accompanied by significant increases in AQP4^+^ area and colocalization ratio relative to the control IgG group (Fig. 2, d-e). Additionally, our results show a similar trend in the penetrating vessels, where increased astrocytic GFAP^+^ area and intensity are highly associated with vascular amyloid deposits in 3D6 treated PDAPP mice, accompanied by significant increases in AQP4^+^ area and colocalization ratio relative to control IgG (Fig. 2, f-j), indicating a potential compensatory increase in barrier responses by glia limitans.

### Spatial proteomics identifies activated T-cells linked to smooth muscle cell loss and macrophages associated with fibrotic responses following Aβ immunotherapy

**Fig. 3:**
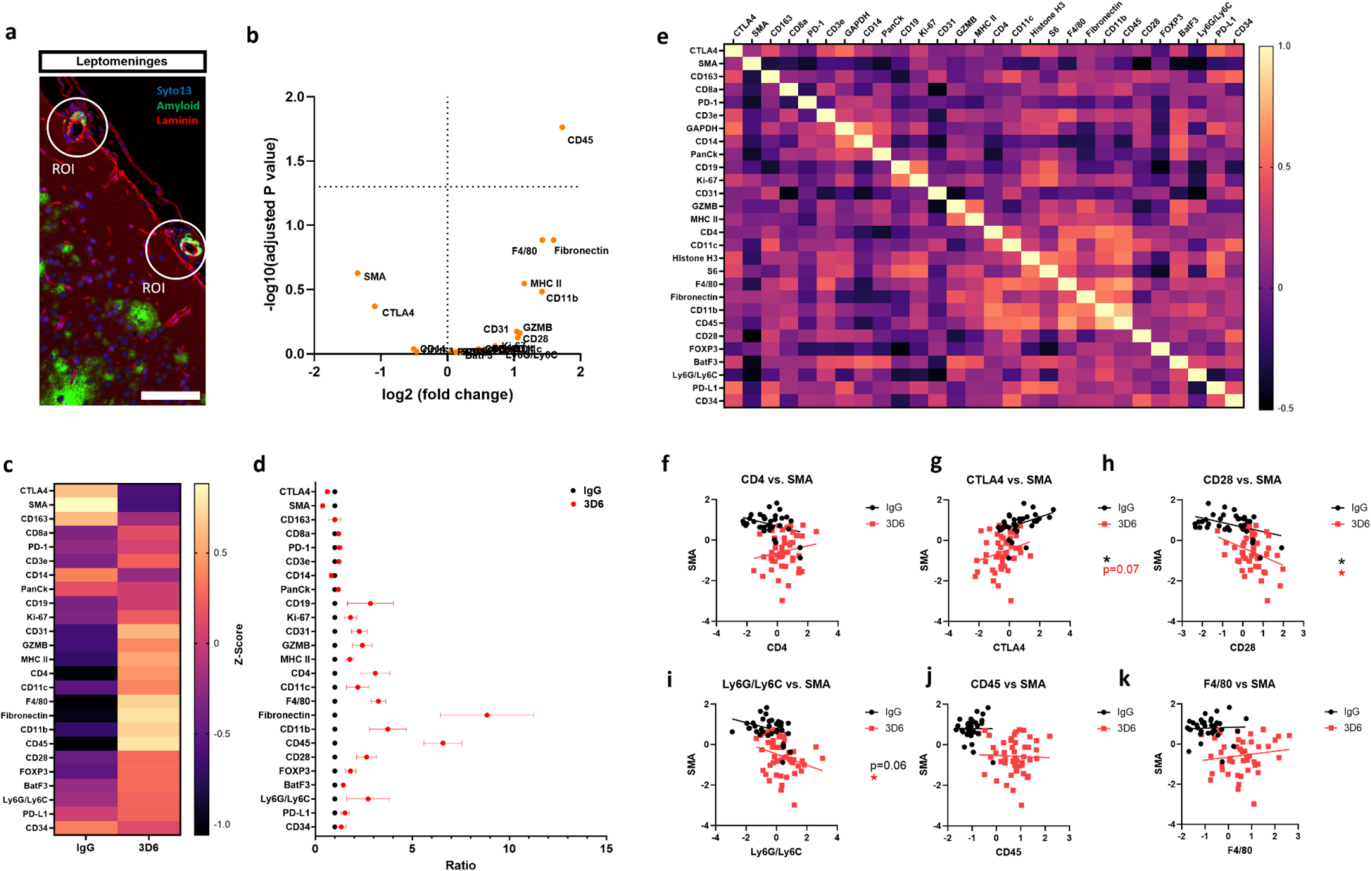
Activated T-cells are linked to smooth muscle cell loss around leptomeningeal vascular amyloid deposits in 3D6 treated PDAPP Mice. **a** 100 μm ROI selections of vascular amyloid deposits in the leptomeninges of PDAPP mice. Triple immunofluorescence of nuclei (blue), amyloid (green) and laminin (red). **b** Volcano plots showing differentially expressed proteins in PDAPP mice treated with 3D6. **c** GeoMx DSP generated heatmap of protein expression (yellow = upregulation, purple = downregulation). **d** Ratio builder of relative expression of target probes. **e** Correlation heatmap of protein expression. **f** Correlation of CD4 and SMA from individual vessels. **g** Correlation of CTLA4 and SMA from individual vessels. **h** Correlation of CD28 and SMA from individual vessels.** i** Correlation of Ly6G/Ly6C and SMA from individual vessels. **j** Correlation of CD45 and SMA from individual vessels. **k** Correlation of F4/80 and SMA from individual vessels. In total 7 mice (IgG treated *n* = 3 and 3D6 treated *n* = 4) were analyzed using NanoString GeoMx™ DSP technology representing 12 CAA containing leptomeningeal vessels per animal. Asterisks indicate significant differences, where *p < 0.05, by unpaired Student’s t test. Scale bar 100 μm

To dissect the intricacies of vascular inflammation in response to Aβ immunotherapy we used NanoString Technologies' GeoMx DSP to evaluate the expression of 25 proteins identified in immune cell profiling and immune cell typing. To focus our investigation, we selected 12 vascular amyloid^+^ regions of interest (ROIs) per animal from either the leptomeninges or penetrating vessels, with each ROI represented by a 100 μm circle. In the penetrating vessels where microhemorrhages are less frequent in this model, no significant differential expression of proteins is observed (Supplemental Fig. 3, a-b). Despite a significant correlation of proteins revealed by Pearson correlation analysis within individual blood vessels, the lack of global differential expression in these vessels is particularly notable in the absence of vascular damage (Supplemental Fig. 3, a-k). In contrast, CD45, a marker for leukocytes, emerged as a key protein exhibiting significant differential expression specifically in the leptomeninges, the primary site of microhemorrhages in PDAPP mice, indicating robust immune activation following 3D6 treatment (Fig. 3, a-b). Due to high degree of variability among individual vessels evaluated, CD45 was the only protein reaching statistical significance however, the relative expression of target probes revealed valuable insights into the underlying immune responses. Following Aβ immunotherapy, SMA undergoes the largest reduction in fold change and relative expression among target probes, while the endothelial cell marker CD31 experiences a slight enrichment (Fig. 3, b-d). These observations are consistent with our confocal analysis results demonstrating loss of SMA, the preservation of endothelial cell and increased vascular endothelial growth factors (Fig. 1, b-j). Furthermore, T-cell markers, Cytotoxic T-lymphocyte associated protein 4 (CTLA4), Granzyme B (GZMB), CD28, and the antigen presentation machinery marker Major histocompatibility complex class II (MHC-II) show differential expression after 3D6 treatment (Fig. 3, b-d), suggesting an adaptive immune response following Aβ immunotherapy. To better understand the interplay between immune responses and vascular damage, we performed Pearson correlation analysis within individual blood vessel examined (Fig. 3, e). While total CD4 protein expression did not correlate with reduction of SMA following Aβ immunotherapy, we observed that SMA protein levels are positively correlated with T-cell inhibitory receptor CTLA4 and inversely correlated with T-cell activating receptor CD28 (Fig. 3, f–h), suggesting T-cell activation is likely involved in the process of smooth muscle loss. Moreover, we also found an inverse correlation between SMA and peripheral monocytes/neutrophils marker Ly6G/C (Fig. 3, i) but not resident macrophage markers such as F4/80 and leukocyte marker CD45 (Fig. 3, j and k), suggesting that peripheral immune infiltration and adaptive immunity, rather than resident macrophages, may contribute to smooth muscle loss.

**Fig. 4:**
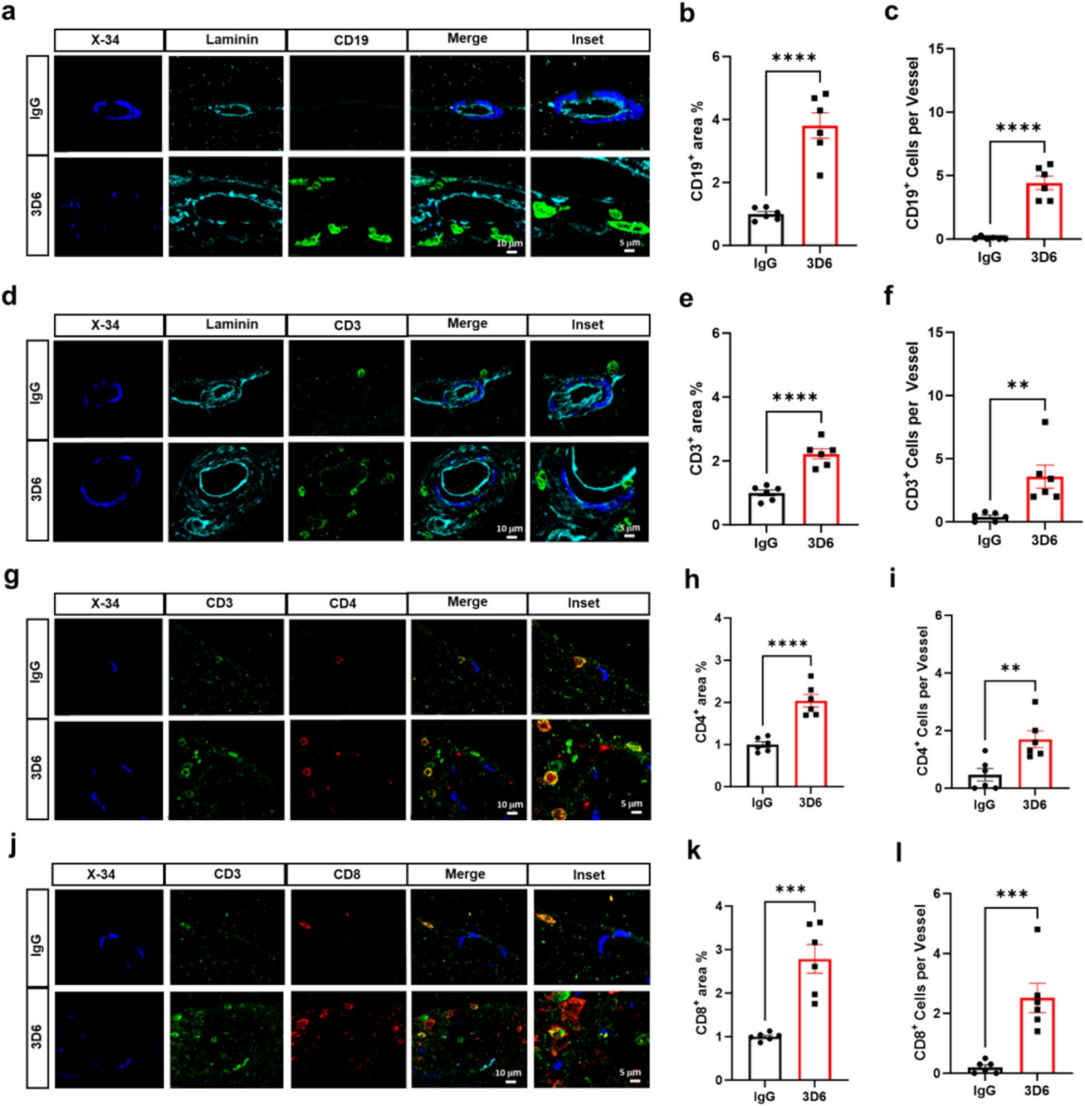
Increased B and T-lymphocytes surrounding vascular amyloid deposits in 3D6-treated PDAPP mice. **a** Triple immunofluorescence of amyloid (X-34, blue), laminin (cyan) and B-cells (CD19, green) in PDAPP mice treated with 3D6 or IgG control. X-34, laminin and CD19 immunoreactivity overlay (Merge). **b** Quantification of CD19^+^ area (%) in IgG or 3D6 treated PDAPP mice. **c** Quantification of CD19^+^ cells per vessel in IgG or 3D6 treated mice. **d** Triple immunofluorescence of amyloid (X-34, blue), laminin (cyan) and T-cells (CD3, green) in PDAPP mice treated with 3D6 or IgG control. X-34, laminin and CD3 immunoreactivity overlay (Merge). **e** Quantification of CD3^+^ area (%) of IgG or 3D6 treated PDAPP mice. **f** Quantification of CD3^+^ cells per vessel in IgG or 3D6 treated PDAPP mice. **g** Triple immunofluorescence of amyloid (X-34, blue), CD3 (green) and CD4 (red) in PDAPP mice treated with 3D6 or IgG control. X-34, CD3 and CD4 immunoreactivity overlay (Merge). **h** Quantification of CD4^+^ area (%) in IgG or 3D6 treated PDAPP mice. **i** Quantification of CD4^+^ cells per vessel in IgG or 3D6 treated PDAPP mice. **j** Triple immunofluorescence of amyloid (X-34, blue), CD3 (green) and CD8 (red) in PDAPP mice treated with 3D6 or IgG control. X-34, CD3 and CD8 immunoreactivity overlay (Merge). **k** Quantification of CD8^+^ area (%) in IgG or 3D6 treated PDAPP mice. **l** Quantification of CD8^+^ cells per vessel in IgG or 3D6 treated PDAPP mice. The number of vascular amyloid deposits analyzed was 8–10 per animal. Results are shown as mean ± SEM of subgroup analysis *n* = 6 (mice). Asterisks indicate significant differences, where ** *p* < 0.01, *** *p* < 0.001 and **** *p* < 0.0001 by unpaired Student’s t test. Scale bar 5 μm or 10 μm, respectively

To confirm the involvement of adaptive immune cells following Aβ immunotherapy, sections were analyzed for the presences of B- and T-lymphocytes. In 3D6-treated PDAPP mice, B-cells are highly abundant around vascular amyloid deposits, with significant increases in CD19^+^ area and CD19^+^ cells per vessel compared to control IgG (Fig. 4, a-c). Moreover, Aβ immunotherapy is also strongly linked to T-cell infiltration, with a significant increase in CD3^+^ area and CD3^+^ cells per vessel relative to control IgG (Fig. 4, d-f) validating our spatial proteomics data (Fig. 3). To further identify the subset of T-cells encircling vascular amyloid deposits, we analyzed helper T-cell marker CD4 and cytotoxic T-cell marker CD8. Our findings show that Aβ immunotherapy is highly associated with both increased CD4^+^ area and CD4^+^ cells per vessel and CD8^+^ area and CD8^+^ cells per vessel relative to control IgG groups (Fig. 4, g-l). To determine if infiltrating lymphocytes are present in an acute/subacute timepoint before the occurrence of microhemorrhages, we employed an additional mouse model of AD. We observed limited infiltration of lymphocytes around vascular amyloid deposits in 24-month-old hTau APP KI mice treated with 3D6 for a duration of 1 month, a time point with robust immune complex formation but limited vascular damages or microhemorrhages (Supplemental Fig. 4, a-f). These findings strongly suggest the recruitment and activation of lymphocytes is linked to vascular damage and accelerated in the context of microhemorrhages, although the precise roles of activated T-cells in microhemorrhage induction remain unclear.

**Fig. 5:**
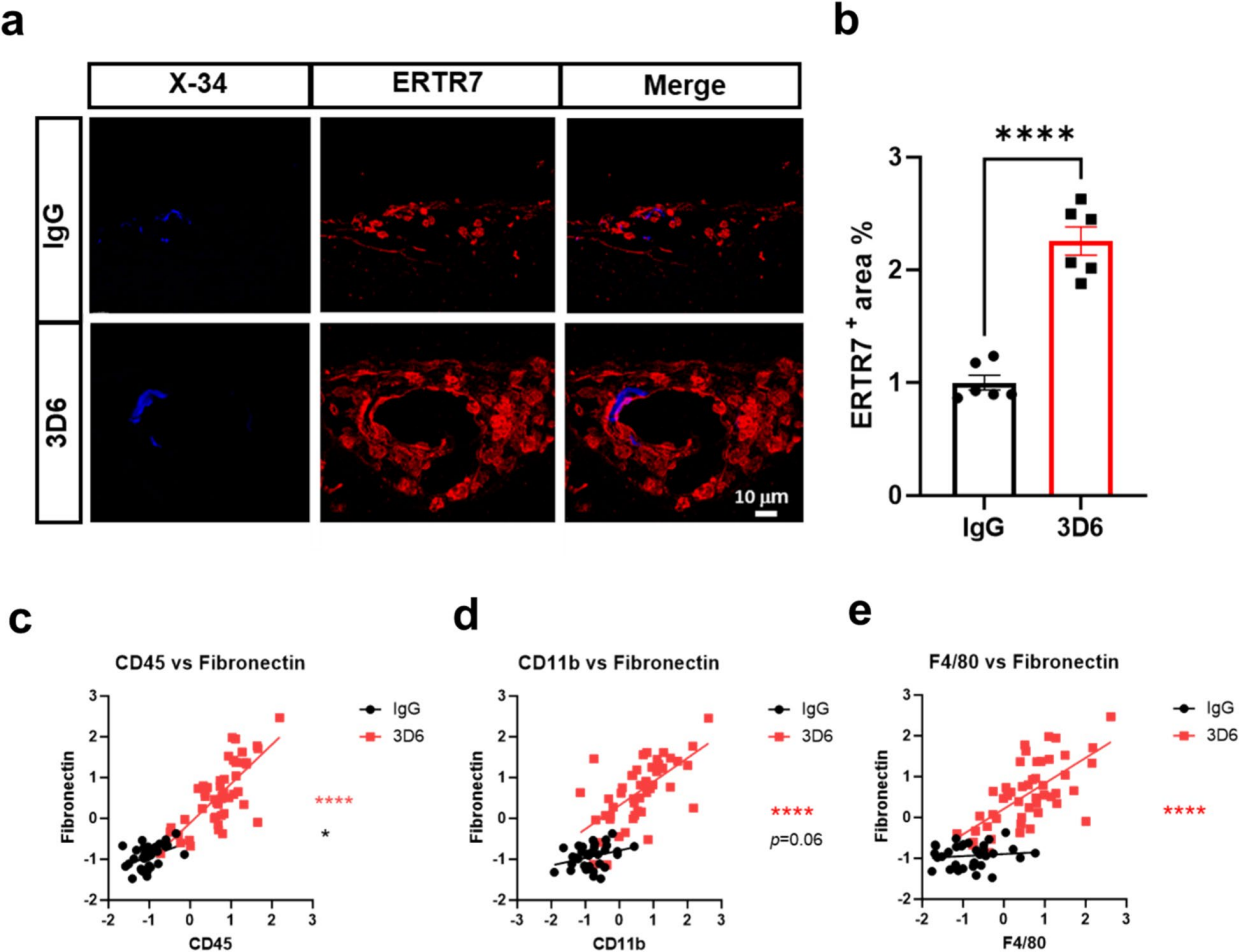
Increased fibrotic response around vascular amyloid deposits in 3D6 Treated PDAPP mice. **a** Double immunofluorescence of amyloid (X-34, blue) and fibroblasts (ERTR7, red) in PDAPP mice treated with 3D6 or IgG control. X-34 and ERTR7 immunoreactivity overlay (Merge). **b** Quantification of ERTR7^+^ area (%) in IgG or 3D6 treated PDAPP mice. Results are shown as mean ± SEM of *n* = 6 (mice). **c** Correlation of CD45 and Fibronectin from individual vessels. **d** Correlation of CD11b and Fibronectin from individual vessels. **e** Correlation of F480 and Fibronectin from individual vessels. Asterisks indicate significant differences, where *p < 0.05 and **** *p* < 0.0001 by unpaired Student’s t test. Scale bar 10 μm

GeoMX DSP also indicated that fibronectin, a marker for fibroblasts, was markedly elevated following Aβ immunotherapy and closely clustered in the correlation heat map with immune markers CD45, CD11b and F4/80 (Fig. 3, e). In 3D6 treated PDAPP mice, fibroblasts were highly concentrated around vascular amyloid deposits, suggesting fibrotic responses following Aβ immunotherapy (Fig. 5, a-b). Interestingly, we observed a significant positive correlation between the immune markers CD45 and CD11b with fibroblasts in IgG-treated animals, which was further amplified following Aβ immunotherapy (Fig. 5, c-d). Importantly, we observed a significant positive correlation between the macrophage marker F4/80 and fibroblasts only in 3D6 treated PDAPP mice (Fig. 5, e) consistent with a role of macrophages in inducing fibrotic responses within injured sites [17].

### *Trem2*^+^*resident and monocyte-derived macrophages are highly associated with vascular amyloid deposits following Aβ immunotherapy*

**Fig. 6:**
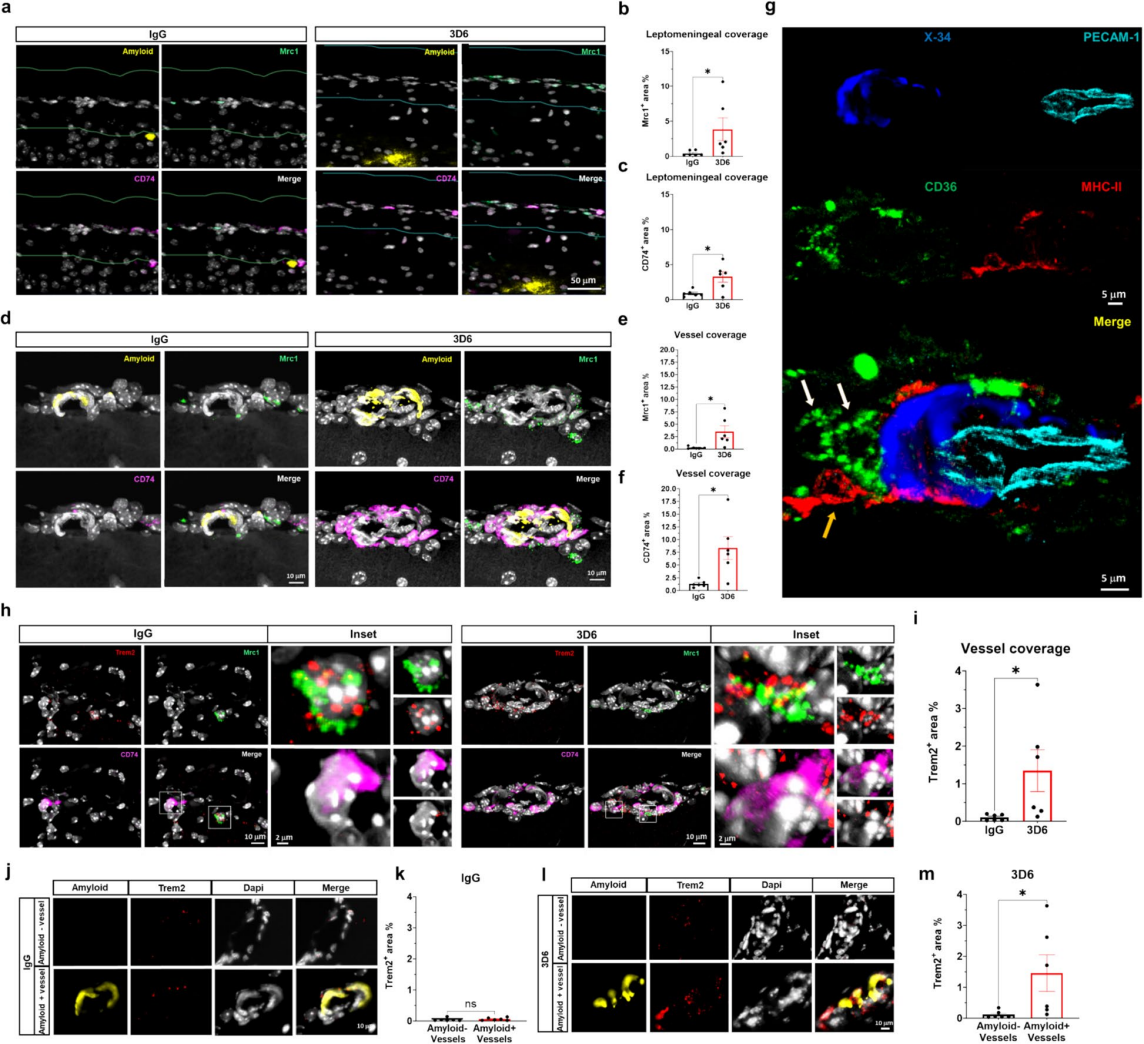
Amyloid immunotherapy is highly associated with increased *Trem2* + macrophages in 3D6 treated PDAPP Mice. **a** Dual RNAscope in situ hybridization of tissue-resident macrophages (Mrc1, green), monocyte-derived macrophages (CD74, purple) and immunofluorescence of amyloid (yellow) across the leptomeninges (colored lines) in PDAPP mice treated with 3D6 or IgG control. Amyloid, Mrc1 and CD74 overlay (Merge). **b** Quantification of leptomeningeal Mrc1^+^ area (%) of IgG or 3D6 treated PDAPP mice. **c** Quantification of leptomeningeal CD74^+^ area (%) in IgG or 3D6 treated PDAPP mice. **d** Dual RNAscope in situ hybridization of tissue-resident macrophages (Mrc1, green), monocyte-derived macrophages (CD74, purple) and immunofluorescence of amyloid (yellow) around amyloid^+^ vessels in PDAPP mice treated with 3D6 or IgG control. Amyloid, Mrc1 and CD74 overlay (Merge). **e** Quantification of Mrc1^+^ area (%) of amyloid^+^ vessels in IgG or 3D6 treated PDAPP mice. **f** Quantification of CD74^+^ area (%) of amyloid^+^ vessels in IgG or 3D6 treated PDAPP mice. **g** Four-color immunofluorescences of amyloid (X-34, blue), endothelial cells (Pecam-1, cyan), tissue-resident macrophages (CD36, green, white arrow) and monocyte-derived macrophages (MHC II, red, yellow arrow) around vascular amyloid deposits in PDAPP mice treated with 3D6. **h** Multiplex RNAscope image of Trem2 (red), tissue-resident macrophages (Mrc1, green) and monocyte-derived macrophages (CD74, purple) around vessels in PDAPP mice treated with 3D6 or IgG control. Trem2, Mrc1 and CD74 immunoreactivity overlay (Merge). **i** Quantification of Trem2^+^ area (%) of vessels in IgG or 3D6 treated PDAPP mice. The number of vessels analyzed was 20. **j** Triple immunofluorescence of amyloid^+^ (yellow) or amyloid^−^ vessels, Trem2 (red) and dapi in PDAPP mice treated with IgG control. Amyloid, Dapi, Trem2 immunoreactivity overlay (Merge). **k** Quantification of Trem2^+^ area (%) of IgG in amyloid^+^ or amyloid^−^ vessels. **l** Triple immunofluorescence of amyloid^+^ (yellow) or amyloid^−^ vessels, Trem2 (red) and dapi in PDAPP mice treated with 3D6. **m** Quantification of Trem2^+^ area (%) of 3D6 in amyloid^+^ or amyloid^−^ vessels. The number of vessels analyzed was 3–7 per animal. Results are shown as mean ± SEM of *n* = 6 (mice). Asterisks indicate significant differences, where *p < 0.05, by unpaired Student’s t test. Scale bar 50 μm, 10 μm, 5 μm or 2 μm respectively

PVMs are a subset of border-associated macrophages (BAMs) in the central nervous system (CNS). Recently fate mapping studies have revealed two distinct subsets of BAMs: monocyte derived BAM-1 are characterized by the expression of MHC-II genes notably *Cd74*, whereas BAM-2 are predominantly yolk-sac derived and express tissue resident macrophage markers such as *Mrc1* [18]. To gain deeper insights into the potential heterogeneity of PVM subsets following Aβ immunotherapy, we examined the abundance of mRNA markers highly indicative of BAM subsets. To determine the spatial distribution of these populations throughout brain, we quantified the percentage area covered by target probes within 100 μm diameter regions across the leptomeningeal surface or around vascular amyloid deposits. Significant increases in both *Mrc1*^+^ and *CD74*^+^ area in the leptomeninges were observed in 3D6-treated PDAPP compared to IgG control, indicating responses from both monocyte-derived (BAM-1) and tissue-resident (BAM-2) macrophages following Aβ immunotherapy (Fig. 6, a-c). Specifically, we found that the both *Mrc1* and *CD74* mRNA transcripts are enriched around vascular amyloid deposits (Fig. 6, d-f). It is interesting to note distinct cellular localization patterns of *Mrc1* and *Cd74* mRNA transcripts with *Mrc1* displaying a pronounced cytoplasmic localization, while *Cd74* appears predominantly nuclear. These findings were further corroborated through immunofluorescence analysis of protein markers for BAM-1 and BAM-2 subsets, namely MHC II and CD36, respectively (Fig. 6, g) and are consistent with our confocal analysis confirming a significant increase in MHC II^+^ area in 3D6-treated PDAPP mice compared to the control IgG group (Supplemental Fig. 5, a and b). In light of recent clinical data highlighting the role of TREM2-agonists antibodies in exacerbating ARIA in AD patients, we sought to investigate the expression of *Trem2* in these macrophage subsets [10]. Notably, our results indicate that Aβ immunotherapy leads to a substantial increase in *Trem2*^+^ area around vessels, with the presence of *Trem2* transcripts identified in both BAM-1 and BAM-2 (Fig. 6, h-i). Importantly, the *Trem2*^+^ area was enriched exclusively around amyloid-positive vessels in 3D6-treated PDAPP mice (Fig. 6, j-m), consistent with a role of TREM2 mediated immune responses to vascular amyloid deposits following Aβ immunotherapy.

## Discussion

Amyloid-related imaging abnormalities (ARIA) have emerged as the most serious adverse events following Aβ immunotherapy, significantly impacting cerebrovascular health. Consistent with our prior findings [9], a strong association between PVM activation and the development of microhemorrhages has been observed in patients who succumbed to ARIA-related events in the Lecanemab clinical trial [7, 8]. In our current investigation, we have furthered our understanding of the consequences of PVM activation following Aβ immunotherapy, uncovering multiple aspects of cerebrovascular damage, including compromised BBB integrity, profound smooth muscle cell loss and vascular fibrosis. The use of multiplexing GeoMx DSP has allowed us to examine changes in multiple immune protein markers within individual blood vessels, revealing that cerebrovascular damage is associated with distinct immune activations.

First, we found that smooth muscle cell loss is not associated with overall PVM responses but is strongly linked to peripheral immunity, particularly T-cell activation. Notably, following Aβ immunotherapy, activated T-cells were localized around vascular amyloid deposits and correlated to smooth muscle cell loss, suggesting a potential connection between peripheral immune cell infiltration and increased vascular permeability. Interestingly, a significant association between T-cell activation and smooth muscle cell loss was also observed in IgG-control treated PDAPP mice (Fig. 3, g and h), indicating that these immune responses occur prior to Aβ immunotherapy, likely triggered by the deposition of CAA. Additionally, the increased expression of MHC II highlighted the active involvement of antigen presentation around vascular amyloid deposits, suggesting a potential role of CD4 T-cells in vascular inflammation associated with smooth muscle loss. These findings suggest two possibilities: infiltrating immune cells may accelerate smooth muscle cell loss, or conversely, smooth muscle cell loss may facilitate immune cell infiltration. Indeed, the occurrence of vascular inflammation and smooth muscle cell loss are prevalent features documented in the progression of CAA cases, with amyloid gradually replacing smooth muscle cells along vessel walls leading to severe degenerative changes associated with cerebral hemorrhage [19, 20]. These results underscore the need for comprehensive evaluation of CAA induced vascular damage in AD, as a key risk factor for ARIA. Unlike smooth muscle cells, endothelial cells are remarkably well preserved even in severe cases of CAA [14, 21]. In alignment with these findings, our data also demonstrate the overall preservation of endothelial cells and, intriguingly, increased endothelial VEGF expression after Aβ immunotherapy. Additionally, we observed increased AQP4 and GFAP expression, indicating a compensatory increase in the barrier response by the glia limitans following Aβ immunotherapy. The precise role of VEGF and AQP4 in regulating the induction or resolution of microhemorrhages, vascular damage, edema or ARIA-E at this stage requires further investigation.

Furthermore, GeoMx DSP revealed the upregulation of fibrotic responses following Aβ immunotherapy mediated vascular injury. The fibrosis marker fibronectin was highly enriched and clustered with immune markers CD45, CD11b, and notably the macrophage marker F4/80, consistent with the role of macrophages in inducing tissue fibrosis [22]. The role macrophages in tissue repair, regeneration, and fibrosis has been extensively studied [17], highlighting their crucial immunoregulatory function in shaping cellular and molecular interactions that influence the fibrotic response. While initially beneficial for wound healing, this process can become maladaptive if it leads to excessive extracellular matrix (ECM) deposition, creating a feedback loop that sustains inflammation and promotes vascular stiffening and reduced compliance [22]. The balance between fibrotic responses and the cross-talk between macrophages and fibroblasts could therefore play a critical role in understanding the pathological remodeling of the vasculature and the potential long-term consequences of fibrosis, hardening and scarring of blood vessels following Aβ immunotherapy-related vascular damage.

BAMs are macrophages situated at various CNS interfaces (such as the dura mater, subdural meninges, leptomeninges, perivascular Virchow-Robin spaces, and choroid plexus). Fate mapping studies indicate that BAMs undergo partial dilution by bone marrow-derived macrophages postnatally, reflecting monocytic input, turnover, and replacement at steady state [18]. In our study, RNA in situ hybridization revealed local accumulation of PVMs around vascular amyloid deposits after Aβ immunotherapy consists of two distinct subsets: those of monocytes derived (*CD74*^+^*,* BAM-1) and tissue-resident (*Mcr1*^+^*,* BAM-2). While the functional difference between BAM-1 and BAM-2 remains unclear, it is intriguing that BAM-1 express high levels of MHC-II and other antigen presentation machinery, suggesting a potential important role in modulating CD4 T-cell immunity during Aβ immunotherapy. TREM2 is an activating receptor expressed in many macrophage populations, including microglia, and has been recognized for its protective role in limiting amyloid deposition and toxicity [23]. In this study, we have demonstrated that both BAM-1 and BAM-2 around vascular amyloid deposits express high level of *Trem2* following Aβ immunotherapy. This observation is particularly significant in light of recent clinical data linking TREM2-agonist antibodies to increased vascular damage and ARIA in Alzheimer's disease (AD) patients [10], suggesting a potential role for TREM2 in regulating microhemorrhages and ARIA. To this extent our confirmation of increased *Trem2*^+^ area around vessels and *Trem2* expression in both PVM subsets provides a plausible explanation for increased ARIA in AD patients and establishes a significant connection between Aβ immunotherapy, vascular damage, compromised BBB integrity, and the recruitment of peripheral and vascular resident immune cells to vascular amyloid deposits; therefore, caution should be exercised when considering the combination of amyloid and TREM2 immunotherapies.

Although both ARIA-H and ARIA-E in AD patients involve immune activation and vascular changes, their underlying mechanisms may differ. Our model resembles ARIA-H, characterized by direct vascular damage and bleeding, potentially driven by macrophages, T-cell activation and smooth muscle cell loss. In contrast, understanding ARIA-E is more challenging due to the limited availability of mouse models but suggests immune activation and vascular changes could be linked to BBB breakdown and fluid leakage. This process is likely mediated by the recruitment of macrophages and the release of matrix metalloproteinases (MMPs) and other enzymes that degrade the extracellular matrix and compromise the BBB. Unlike ARIA-H, which involves overt bleeding, ARIA-E may result from more subtle leakage, leading to fluid accumulation without significant red blood cell extravasation.

Overall, our work suggests that the formation of 3D6 immune complexes with vascular amyloid deposits initiates an immune response cascade, likely beginning with tissue-resident macrophages interacting with these complexes to create an inflammatory environment that recruits peripheral immune cells. Activation of T-cells and potentially granulocytes appears to play a critical role, as they are closely linked to the loss of smooth muscle cells, which may increase vascular permeability and exacerbate vascular damage, ultimately resulting in microhemorrhage. We also identified a potential role for macrophages in promoting fibrotic responses, suggesting extensive tissue remodeling as part of the wound healing process following microhemorrhage. However, due to the lack of sensitive markers available to detect the rapid and transient dynamics of acute BBB leakage, the precise relationship between vascular changes and sites of antibody binding or acute microbleeds remains elusive. This limitation highlights the need for further research to explore these relationships in greater detail and develop more refined tools for detecting early-stage vascular changes to clarify these precise relationships.

## Conclusions

In summary, our findings establish a significant connection between Aβ immunotherapy, vascular damage, compromised BBB integrity, and the recruitment of both peripheral and vascular resident immune cells to vascular amyloid deposits. This provides compelling evidence that the observed changes are indeed linked to the downstream consequences of macrophage engagement and activation by immune complexes rather than the redistribution of Aβ from the brain parenchyma to the cerebral vasculature. While these insights enrich our understanding of CAA-mediated immune response following Aβ immunotherapy, further investigations are imperative to fully comprehend the implications of this phenomenon on microhemorrhage susceptibility. Specifically, understanding the immediate downstream consequences of macrophage activation and elucidating the specific roles played by tissue-resident and monocyte-derived macrophages are crucial in unraveling the interplay between immune activation and vascular damage triggered by Aβ immunotherapy. Nevertheless, we acknowledge that the specific roles played by resident and monocyte-derived macrophages during Aβ immunotherapy remain incompletely understood. To delve deeper into these differences, conducting further transcriptomic analyses using multiplex spatial transcriptomics, as well as examining human brain samples—particularly those from clinical immunotherapy trials—will be imperative. This approach will allow us to comprehensively dissect the interplay of diverse immune populations and the functional roles of various macrophage populations in the human brain during Aβ immunotherapy, ultimately helping to decipher the precise mechanisms leading to microhemorrhages.

## Methods

### Transgenic mouse model

PDAPP and hTau APP KI male and female mice were used in our experiments, including digital spatial profiling and immunohistochemistry (IHC) analyses. PDAPP colony (line 13,388) was established through an inbreeding exercise wherein mice were inbred from selected litters that maintained decreased variability in both soluble and insoluble Aβ. The plaque deposition phenotype of the inbred PDAPP line 13,388 was similar to the originally described PDAPP colony [11]. The pathology of APP KI mice is characterized by predictable plaque development with progressive increases in parenchymal amyloid pathology as well as mild but detectable cerebral amyloid angiopathy [24]. PDAPP mice received weekly subcutaneous injections of 3D6 (anti-Aβ1-x,IgG2b) (25 mg/kg) or twice-weekly subcutaneous injections of IgG (IgG2a isotype) (50 mg/kg) for three months. hTau APP KI mice received weekly subcutaneous injections of biotynlated-3D6 (anti-Aβ1-x,IgG2b) (25 mg/kg) or weekly subcutaneous injections of IgG (IgG2a isotype) (25 mg/kg) for one month. For dosing experiments, 23-to 26-month-old were utilized. This age was selected due to the extensive vascular amyloid accumulation at 23–26 months of age in the PDAPP and hTau APP KI models. All experiments were performed in accordance with the Institutional Animal Care and Use Guidelines for Eli Lilly.

### Brain sections immunofluorescence

Mice were anesthetized with avertin and perfused with ice-cold PBS. Brains were carefully removed from the cranium to avoid separating the meninges and fixed in 4% (wt/vol) paraformaldehyde (PFA) for 24 h followed by cryoprotection in 30% (wt/vol) sucrose in PBS solution. hTau APP KI brains were subsequently frozen and coronally sectioned (10 μm thick) using a freezing/sliding microtome. PDAPP coronal brain Sects. (10 μm thick) were paraffin embedded, deparaffinized in xylene, rehydrated in ethanol (EtOH) and washed with deionized water. Then, sections were heated in low pH 1 × citrate buffer for 30 min. After washing in PBS for 5 min twice, the sections were blocked with a solution of 5% goat serum and 0.01% Triton X-100 in PBS for 1 h at room temperature (RT). Sections were then incubated overnight at 4 °C with the following antibodies, each diluted 1:100 in blocking solution: anti-Fibrinogen ( PA1-85,429, ThermoFisher), anti-SMA (PA585070, ThermoFisher), anti-CD 31 (14–0311-82, ThermoFisher), anti-VEGF (19,003–1-AP, ThermoFisher), anti-Cldn5 (34–1600, ThermoFisher), anti-GFAP (13–030-0, ThermoFisher), anti-AQP4 (50–173-0968, ThermoFisher), anti-CD19 (14–0194-82, ThermoFisher), anti-CD3 (PIMA514524, Fisher), anti-CD4 (14–9766-82, ThermoFisher), anti-CD8 (14–0808-82, ThermoFisher), anti-Laminin (NB300-144,NovusBio) anti-MHC II (107,610, Citeab), anti-ERTR7 (NB100-64932, Novus) and anti-CD36 (18,836–1-AP, ThermoFisher). The next day, the sections were washed 3 times in PBS and incubated with the following secondary antibodies,1 h at room temperature, diluted 1:100 in blocking solution: goat anti-mouse IgG 555 (A31570, ThermoFisher), goat anti-mouse IgG 647 (A32728,ThermoFisher), goat anti-rabbit IgG 555 (A32732,ThermoFisher), goat anti-rabbit IgG 647 (A32733,ThermoFisher), goat anti-rat IgG 555 (A21434,ThermoFisher), goat anti-rat IgG 647 (A48272,ThermoFisher), streptavidin 488 (S32354, ThermoFisher). Slides were then crosslinked with 4% pfa for 5 min followed by two 5-min washes in deionized PBS. Amyloid was stained with X-34 (SML1954, 1:10,000) prepared in 50% EtOH in TBS for 10 min at RT, followed by two 5-min washes in 50% EtOH and two 5-min washes in PBS. Finally, the sections were washed in PBS and mounted with ProLong Diamond Antifade medium (P36961, ThermoFisher).

### Microhemorrhage analysis

Slides were mounted with six μm thick coronal tissue sections for detection of vascular bleeds marked by hemosiderin deposits by incubating in 2% potassium ferrocyanide (EMS, 26,613–01) in 2% hydrochloric acid for 30 min followed by two 5-min washes in PBS. Slides utilized for microhemorrhage analysis were counter stained with nuclear fast-red (N8002, Sigma-Aldrich) for 5 min followed by a 5-min wash in PBS. Finally, the sections were washed in PBS and mounted with ProLong Diamond Antifade medium (P36961, ThermoFisher). Whole brain slices were scanned using the Zeiss AxioScan Z.1 with a 20X objective (0.8NA). Images were then analyzed for region of interest defined along images of tissue edges (100 μm wide) and Hemosiderin^+^ signal was thresholded to quantify percent coverage of stain relative to ROI using HALO v3.5 image analysis suite (Indica Labs).

### GeoMx digital spatial profiling

Assay was performed on 10 μm thick FFPE (Formalin-fixed paraffin-embedded) coronal brain sections from IgG- and 3D6-treated PDAPP mice. Sections were mounted onto charged slides and underwent baking at 65 °C in a drying oven for 2 h prior to deparaffinization. Slides were deparaffinized and rehydrated by incubating in CitriSolv (Decon Labs) 3 times for 5 min each followed by 2 washes of 100% ethanol for 5 min and 2 washes of 95% Ethanol for 5 min and 2 washes of distilled water for 5 min each. The following steps were conducted on the BOND RXm (Leica Biosystems®, Baden-Wurttemberg, Germany) instrument. Slides were removed from the BOND RXm and were incubated overnight at 4 °C with a multiplexed cocktail of primary antibodies each of which are conjugated to unique ultraviolent-photocleavable oligonucleotide tags (GeoMx Immune Profile Core and Immune Cell Typing modules containing 31 targets, including 6 control probes). Additional fluorescently labeled antibodies were used as morphology markers to select regions of interest (ROIs). In the current study, the morphology markers included β-amyloid (MOAB-2, Novus Biologicals, NBP2-13075AF594), and laminin (Novus Biologicals, NB300-144AF532) to visualize β-amyloid deposition and neuro-vasculature, respectively. Following overnight incubation, slides were washed 3 times in 1X tris-buffered saline with Tween-20 (TBS-T) for 10 min each. Slides were then post-fixed in 4% paraformaldehyde (ThermoFisher, FB002, R37814) for 30 min at room temperature followed by two washes of TBS-T for 5 min each. For nuclear staining, slides were incubated in 1:10 dilution of Nuclear Stain Morphology Kits (DNA dye, SYTO 13), for 15 min at room temperature. Slides were washed with TBS-T for 5 min and loaded onto the GeoMx™ DSP according to the manufacturer’s instructions. In total, 7 mice (IgG treated *n* = 3 and 3D6 treated *n* = 4) were analyzed using NanoString GeoMx™ DSP technology. For each mouse, 12 CAA containing leptomeningeal and 12 CAA containing penetrating vessels were selected as ROIs for high-resolution multiplex profiling, comprising a total of 24 ROIs per animal. A 100-μm in diameter circle was selected as the center ROI surrounding each blood vessel with β-amyloid deposition. The specific 100 μm ROIs for molecular profiling were then sequentially processed by the microscope automation, as previously described [25–27]. In brief, ROIs were selectively illuminated with UV light to release the indexing oligos by coupling UV LED light with a double digital mirror device module. Following each UV illumination cycle, the eluent was collected from the defined region and transferred to an individual well of a microtiter plate. Once all ROIs were processed, pools of released indexing oligos were hybridized to NanoString optical barcodes for digital counting based on the manufacturer's directions. Hybridizations were performed at 65 °C overnight in a thermocycler. After hybridization, samples were processed using the nCounter Prep Station and Digital Analyzer as per manufacturer instructions. Data were normalized to technical controls and areas. Digital counts were normalized with negative controls (Rt IgG2b, Rb IgG, and Rt IgG2a).

### *RNAscope *in situ* hybridization*

In short, 5 μm thick FFPE fixed coronal mounted tissue sections were serially dehydrated in 50%, 70%, 95%, 100% and 100% ethanol for 5 min each. In between all pretreatment steps, tissue sections were briefly washed with ultra-pure water. Incubation, wash, and probe application periods were performed by utilizing the RNAscope® LS Multiplex Fluorescent Reagent kit system (Advanced Cell Diagnostics) and RNAscope® LS 4-plex Ancillary Kit for Multiplex Fluorescent Reagent (Advanced Cell Diagnostics) on the BOND RX (Leica). Mounted slices were treated with pretreat solution 3 (protease reagent) for 15 min at 40 °C. Slides were then incubated with custom mouse CD74 RNAscope® probes (catalog #437,508-C4, ACD), Mrc1 (Catalog #437,518-C1, ACD) Trem2 (Catalog #404,118-C2, ACD). Following in situ hybridization, the sections were processed for immunohistochemistry. Briefly, following the blocking step with 5% normal goat serum (NGS) in PBS for 1 h at room temperature, post-hybridized slides were incubated 1:100 dilution with a 555-fluorescent conjugated antibody (NBP2-13075R, Novus) or 647-fluorescent conjugated antibody (NBP2-13075AF647, Novus) against amyloid in the presence of 2% NGS in PBS overnight at 4 °C. Brain sections were rinsed with PBS three times and incubated for 5 min in PBS with DAPI solution (1:50,000) for counterstained nuclei. Whole brain slices were scanned using the Zeiss AxioScan Z.1 with a 20X objective (0.8NA). Images were then analyzed for region of interest defined around vessels (100 μm circle) or along tissue edges (100 μm wide) and CD74^+^, Mrc1^+^, and Trem2^+^ signal was thresholded to quantify percent coverage of stain relative to ROI using HALO v3.5 image analysis suite (Indica Labs).

### Microscopy and image analysis

For image analysis of mouse brain sections, we used ImageJ software v1.53 (NIH) to create one index that represented changes in the number of Fibrinogen^+^,SMA^+^,PECAM-1^+^, VEGF^+^,Cldn5^+^,GFAP^+^, AQP4^+^, CD19^+^,CD3^+^,CD4^+^, CD8^+^, MHC II^+^,or CD36^+^ pixels divided by the total number of pixels in the image, expressed as ( +) area %. To ensure the representativeness of our study's findings, we applied random selection when choosing animals from the total population for subsequent immunohistological investigations. A subgroup of 3D6 and IgG treated PDAPP mice (6 animals each) was randomly selected across microhemorrhage groups and cerebral cortices were examined using a SP8 confocal microscope (Leica), 63 × objective with a 0.15 μm z-step. We analyzed 8–10 vascular amyloid deposits from both the leptomeninges and penetrating vessels per animal across the entire coronal sections, using two brain sections per mouse. We quantified X-34^+^ area across the leptomeninges, focusing on a 100 μm diameter region along the entire leptomeningeal surface, while the vascular amyloid^+^ area percentage was assessed based on 3D6 immunoreactivity. Furthermore, we quantified the leptomeningeal diameters in microns by measuring from the border of the tissue edge to the pial surface. Results are shown as mean ± SEM of subgroup analysis *n* = 6 (mice). For colocalization quantification, images were analyzed in Imaris v8.4 (Bitplane) to determine relative colocalization coefficients (colocalization ratio) using Manders’ coefficients [28].

### Statistics and reproducibility

The details about experimental design and statistics used in different data analyses performed in this study are given in the respective sections of results and methods. Sample sizes were determined based on previous publications. Significance in NanoString experiments was assessed employing a two-stage linear step-up procedure by Benjamin, Krieger, and Yekutieli through a multiple t test (unpaired t test on each row) [29]. The adjusted p-value threshold was set at alpha 0.05, utilizing the Holm-Sidak correction method. The results are represented as -log10 adjusted *p*-values for clarity. Additionally, ratio builder generated ratios of relative expression of target probes, calculating the ratio of each probe in every segment relative to the average of one or more segments chosen as the baseline. Pearson correlation analysis was used to analyze the relationship between different immune cells in Spotfire software and *P* < 0.05 was considered statistically significant. For imaging experimental modalities utilized in this study, a minimum of six independent biological replicates were employed to ensure robust and reliable results. Investigators were blinded for staining experiments. GraphPad Prism v10 was used to perform all statistical analyses. Statistical significance between groups was calculated using a student’s *t*-test (two-tailed). Data is presented as the mean ± SEM unless otherwise stated. *, **, *** and **** denote *p* < 0.05, *p* < 0.01, *p* < 0.001 and *p* <  < 0.0001, respectively. No other statistical comparisons were significant unless otherwise noted.

## Supplementary Information

Supplementary Material 1.  Supplemental Fig. 1. Reduced amyloid immunoreactivity is associated with vascular damage in 3D6 treated PDAPP mice. (a) Prussian Blue (hemosiderin, blue) labeled microhemorrhages in the leptomeninges of PDAPP mice treated with IgG or 3D6. (b) Quantification of Prussian blue + area (%) in brain coronal sections of IgG or 3D6-treated PDAPP mice. (c) Measurement of the diameter (dashed line) of the leptomeninges in PDAPP mice treated with IgG or 3D6. (d) Correlation of Prussian Blue + area and vascular amyloid immunoreactivity in PDAPP mice treated with IgG. (e) Correlation of Prussian Blue + area and vascular amyloid immunoreactivity in PDAPP mice treated with 3D6. For quantifications, total coronal sections were used, and each data point indicates an animal *n* = 20–25 (mice). (f) Fibrinogen (red) immunoreactivity in the leptomeninges of PDAPP mice treated with 3D6 or IgG control. (g) Quantification of fibrinogen + area (%) of IgG or 3D6 treated PDAPP mice. (h) Amyloid deposits (X-34, blue) in PDAPP mice treated with IgG or 3D6. (i) Quantification of X-34 + area (%) across the leptomeninges in PDAPP mice treated with IgG or 3D6, focusing on a 100 μm diameter region across the entire leptomeningeal surface. (j) Quantification of X-34 + area (%) across the parenchyma of IgG or 3D6 treated PDAPP mice. Results are shown as mean ± SEM of subgroup analysis *n* = 6 (mice), asterisks indicate significant differences, where **p < 0.01, ****p < 0.0001 by unpaired Student's t test. Scale bar 50 and 100 μm respectively.  Supplemental Fig. 2. Claudin-5 is unchanged in 3D6 treated PDAPP mice. (a) Four-color immunofluorescence of amyloid (X-34, blue), fibrinogen (red), claudin 5 (Cldn5, green) and endothelial cells (PECAM-1, cyan) in PDAPP mice treated with 3D6 or IgG control. X-34, Fibrinogen, Cldn5 and PECAM-1 immunoreactivity overlay (Merge). (b) Quantification of Claudin 5 +  area (%) in IgG or 3D6 treated PDAPP mice. (c)  Quantification of Claudin 5 +  intensity of endothelial cells in IgG or 3D6 treated PDAPP mice. The number of vascular amyloid deposits analyzed was 8–10 per animal. Results are shown as mean ± SEM of subgroup analysis *n* = 6 (mice). Scale bar 3 μm.  Supplemental Fig. 3. Peripheral immune cells are not highly recruited around vascular amyloid deposits of penetrating vessels in 3D6 treated PDAPP Mice. (a) 100 μm ROI selections of vascular amyloid deposits in the penetrating vessels of PDAPP mice. Triple immunofluorescence of nuclei (blue), amyloid (green) and laminin (red). (b)  Volcano plots showing directed differentially expressed protein markers in PDAPP mice treated with 3D6. (c) GeoMx DSP generated heatmap of protein expression (yellow = upregulation, purple = downregulation). (d) Ratio builder of relative expression of target probes. (e) Correlation heatmap of protein expression. (f) Correlation of CD4 and SMA from individual vessels. (g) Correlation of CTLA4 and SMA from individual vessels. (h) Correlation of CD28 and SMA from individual vessels. (i) Correlation of Ly6G/Ly6C and SMA from individual vessels. (j) Correlation of CD45 and SMA from individual vessels. (k) Correlation of F4/80 and SMA from individual vessels. In total 7 mice (IgG treated *n* = 3 and 3D6 treated *n* = 4) were analyzed using NanoString GeoMx™ DSP technology representing 12 CAA containing penetrating vessels per animal. Asterisks indicate significant differences, where * *p*  < 0.05, by unpaired Student’s t test. Scale bar 100 μm.  Supplemental Fig. 4. Infiltrating T-cells are not observed after 1 month of 3D6 treatment in hTau APP KI PDAPP mice. (a) Triple immunofluorescence of amyloid (X-34, blue), CD3 (green) and CD4 (red) in hTau APP KI mice treated with 3D6 or IgG control. X-34, CD3 and CD4 immunoreactivity overlay (Merge). (b) Quantification of CD4 + area (%) in IgG or 3D6 treated hTau APP KI mice. (c) Quantification of CD4 + cells per vessel in IgG or 3D6 treated hTau APP KI mice. (d) Triple immunofluorescence of amyloid (X-34, blue), CD3 (green) and CD8 (red) in hTau APP KI mice treated with 3D6 or IgG control. X-34, CD3 and CD8 immunoreactivity overlay (Merge). (e) Quantification of CD8 + area (%) in IgG or 3D6 treated hTau APP KI mice. (f) Quantification of CD8 + cells per vessel in IgG or 3D6 treated hTau APP KI mice. Results are shown as mean ± SEM of *n* = 3 (mice). Scale bar 10 μm.  Supplemental Fig. 5. Increased MHC II around vascular amyloid deposits in 3D6 treated PDAPP mice. (a) Triple immunofluorescence of amyloid (X-34, blue), major histocompatibility complex class II molecules (MHC II, red) and endothelial cells (PECAM-1, cyan) in PDAPP mice treated with 3D6 or IgG control. X-34, MHC II and PECAM-1 immunoreactivity overlay (Merge). (b) Quantification of MHC-II + area (%) in IgG or 3D6 treated PDAPP mice. Results are shown as mean ± SEM of *n* = 6 (mice). Asterisks indicate significant differences, where *** *p* < 0.001 by unpaired Student’s t test. Scale bar 10 μm.

## Data Availability

The NanoString data supporting the conclusions of this article are available at NCBI’s Gene Expression Omnibus (GEO) and is accessible via series accession numbers GSE253388.

## Abbreviations

Aβ: Amyloid-β
AD: Alzheimer disease
APP: Amyloid precursor protein
AQP4: Aquaporin 4
ARIA: Amyloid-related imaging abnormalities
ARIA-E: (Amyloid-Related Imaging Abnormalities with Edema)
ARIA-H: (Amyloid-Related Imaging Abnormalities with Hemorrhage)
A.U.: Arbitrary units
BAM: Border-associated macrophages
BBB: Blood brain barrier
CAA: Cerebral amyloid angiopathy
CNS: Central nervous system
CTLA4: Cytotoxic T-lymphocyte associated protein 4
DSP: Digital spatial profiling
GZMB: Granzyme B
IgG: Immunoglobulin G
MHC II: Major histocompatibility complex 2
PECAM-1: Platelet/endothelial cell adhesion molecule-1
PVM: Perivascular macrophage
SMA: Smooth muscle actin
TREM2: Triggering receptor expressed on myeloid cells 2
VEGF: Vascular endothelial growth factor
