# Supplementary material for: Amyloid-β (Aβ) immunotherapy induced microhemorrhages are linked to vascular inflammation and cerebrovascular damage in a mouse model of Alzheimer’s disease
Source: Mol Neurodegener. 2024 Oct 21;19:77. doi: 10.1186/s13024-024-00758-0 (PMC11494988; doi:10.1186/s13024-024-00758-0)

**Supplemental figure 1**

**
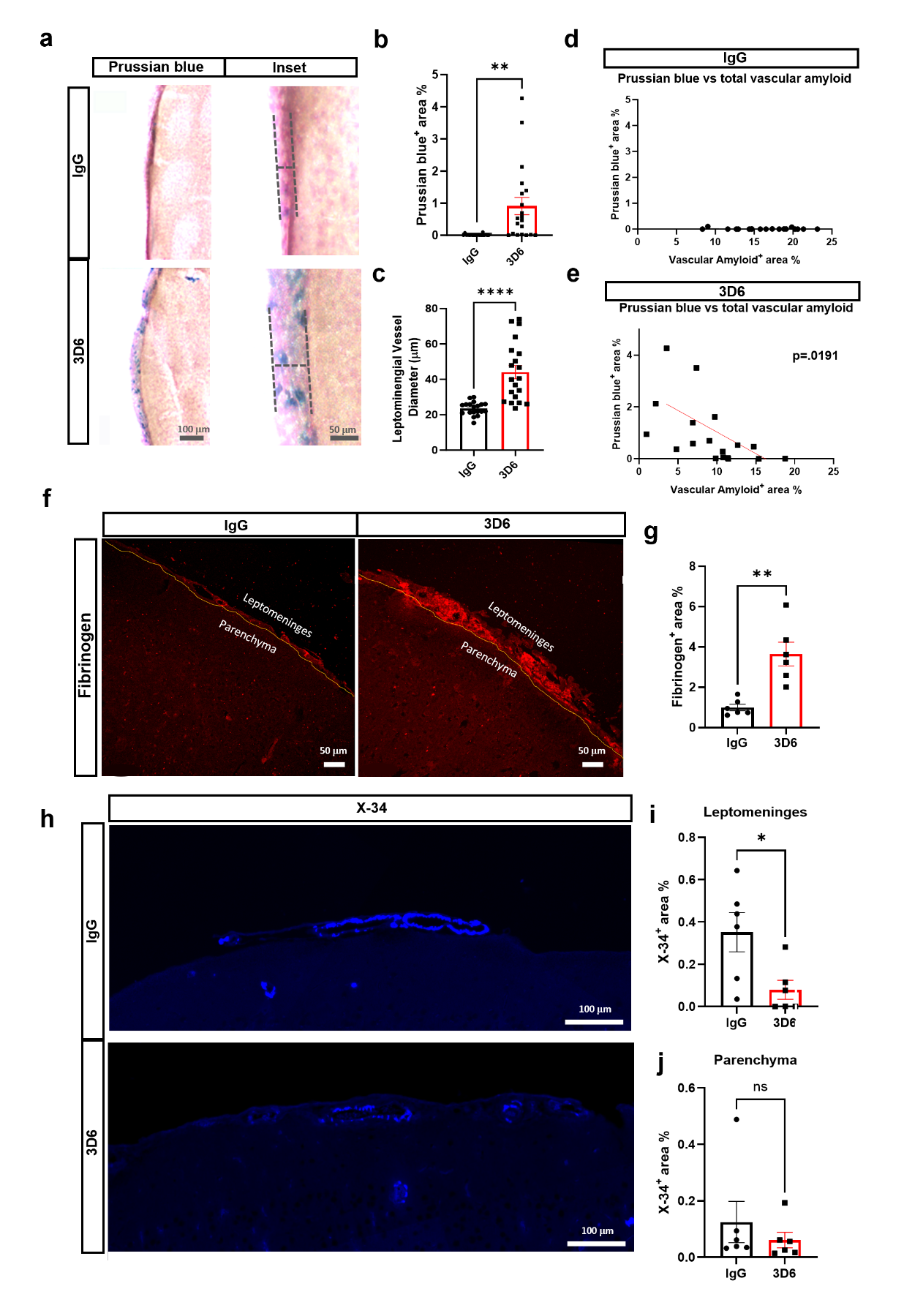
**

**Supplemental figure 2**


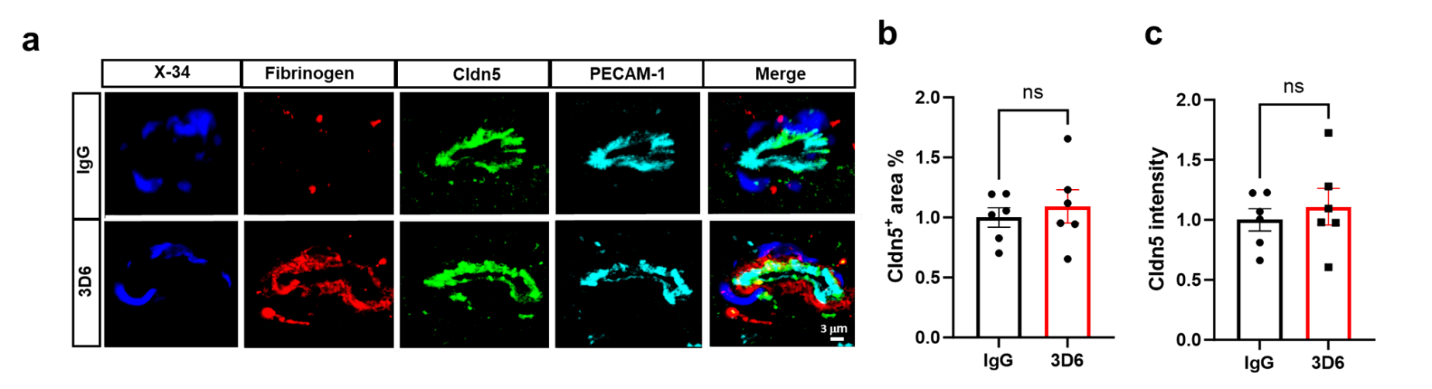


**
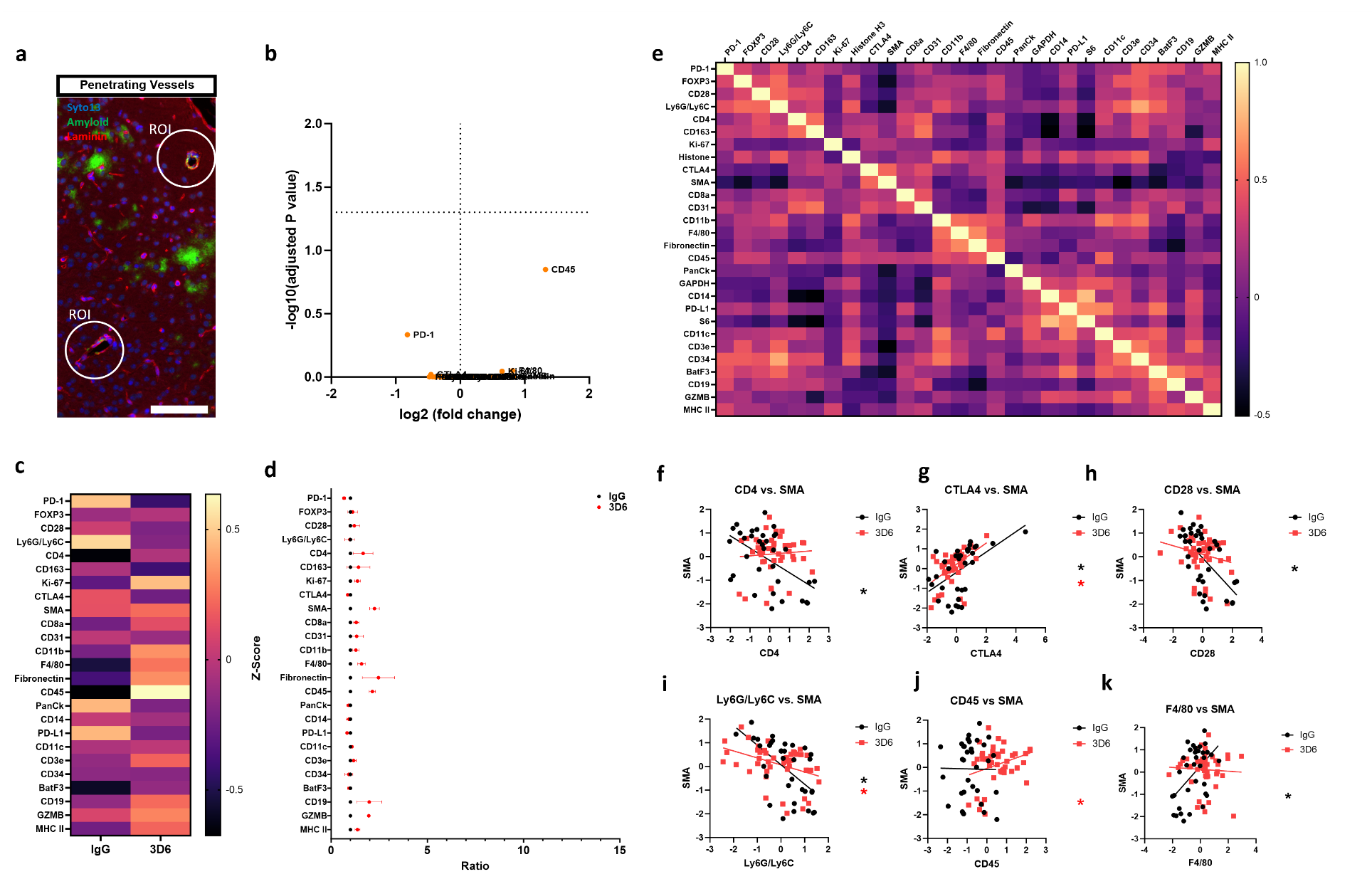
Supplemental figure 3**

**Supplemental figure 4**


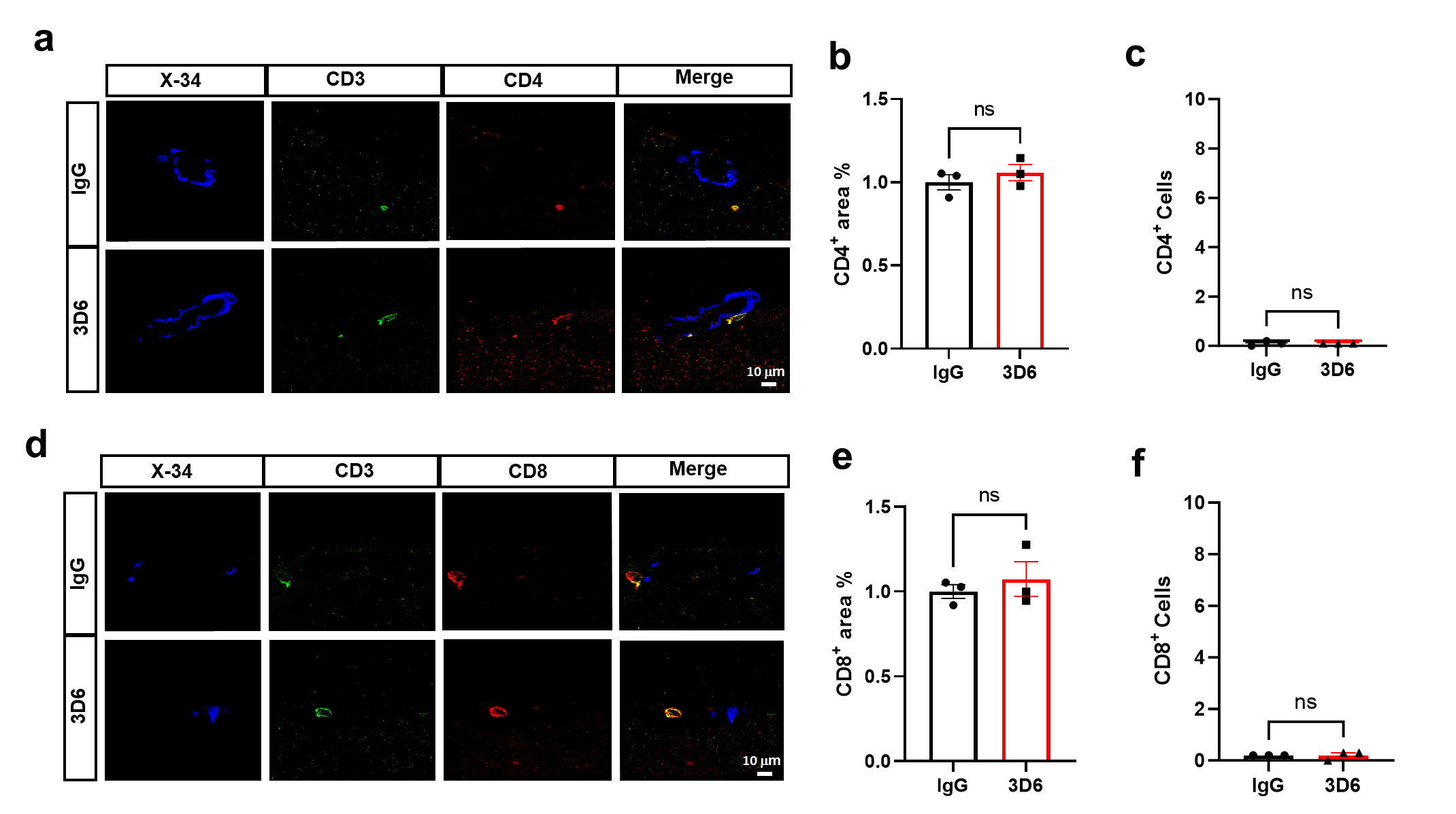


**Supplemental Figure 5**


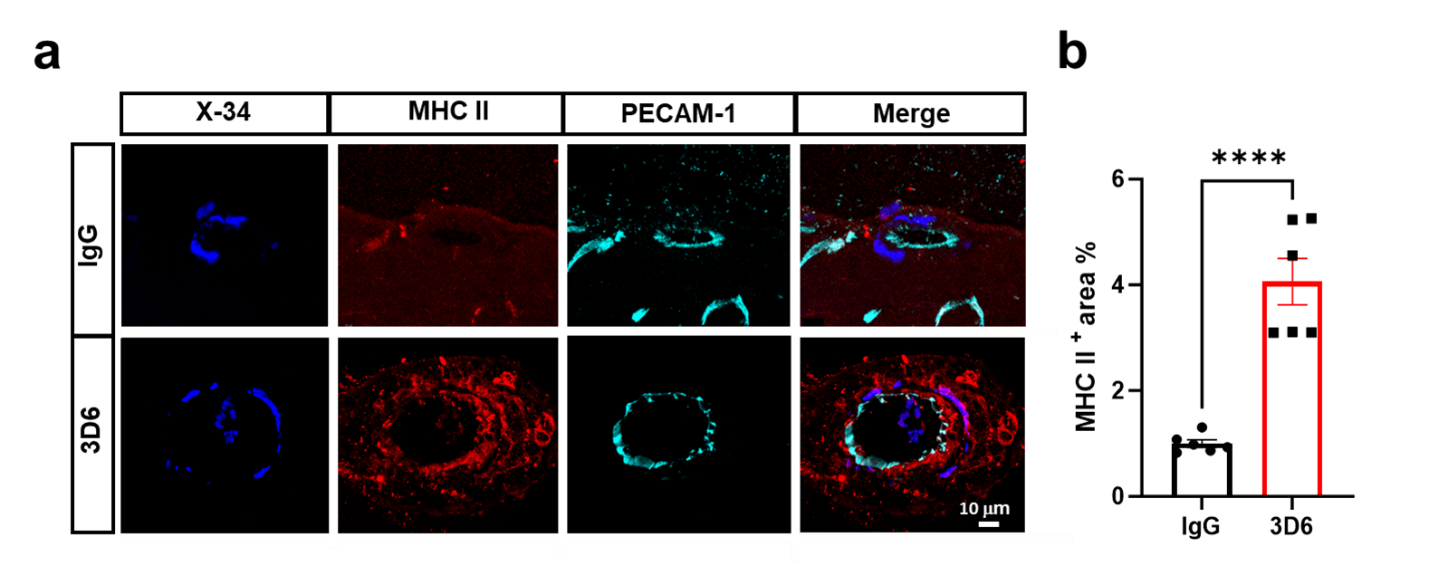

Supplement: Supplementary file 1 — Supplementary Material 1. Supplemental Fig. 1. Reduced amyloid immunoreactivity is associated with vascular damage in 3D6 treated PDAPP mice. (a) Prussian Blue (hemosiderin, blue) labeled microhemorrhages in the leptomeninges of PDAPP mice treated with IgG or 3D6. (b) Quantification of Prussian blue + area (%) in brain coronal sections of IgG or 3D6-treated PDAPP mice. (c) Measurement of the diameter (dashed line) of the leptomeninges in PDAPP mice treated with IgG or 3D6. (d) Correlation of Prussian Blue + area and vascular amyloid immunoreactivity in PDAPP mice treated with IgG. (e) Correlation of Prussian Blue + area and vascular amyloid immunoreactivity in PDAPP mice treated with 3D6. For quantifications, total coronal sections were used, and each data point indicates an animal n = 20–25 (mice). (f) Fibrinogen (red) immunoreactivity in the leptomeninges of PDAPP mice treated with 3D6 or IgG control. (g) Quantification of fibrinogen + area (%) of IgG or 3D6 treated PDAPP mice. (h) Amyloid deposits (X-34, blue) in PDAPP mice treated with IgG or 3D6. (i) Quantification of X-34 + area (%) across the leptomeninges in PDAPP mice treated with IgG or 3D6, focusing on a 100 μm diameter region across the entire leptomeningeal surface. (j) Quantification of X-34 + area (%) across the parenchyma of IgG or 3D6 treated PDAPP mice. Results are shown as mean ± SEM of subgroup analysis n = 6 (mice), asterisks indicate significant differences, where **p < 0.01, ****p < 0.0001 by unpaired Student's t test. Scale bar 50 and 100 μm respectively. Supplemental Fig. 2. Claudin-5 is unchanged in 3D6 treated PDAPP mice. (a) Four-color immunofluorescence of amyloid (X-34, blue), fibrinogen (red), claudin 5 (Cldn5, green) and endothelial cells (PECAM-1, cyan) in PDAPP mice treated with 3D6 or IgG control. X-34, Fibrinogen, Cldn5 and PECAM-1 immunoreactivity overlay (Merge). (b) Quantification of Claudin 5 + area (%) in IgG or 3D6 treated PDAPP mice. (c) Quantification of Claudin 5 + intens [file 13024_2024_758_MOESM1_ESM.docx]
